# Comparison and predictors of chronic migraine vs. new daily persistent headache presenting with a chronic migraine phenotype

**DOI:** 10.1111/head.14362

**Published:** 2022-07-21

**Authors:** Karthik Nagaraj, Diana Y. Wei, Francesca Puledda, Hsing‐Yu Weng, Sadaf Waheed, Nicolas Vandenbussche, Jonathan J. Y. Ong, Peter J. Goadsby

**Affiliations:** ^1^ Headache Group, Wolfson CARD, Institute of Psychiatry, Psychology and Neuroscience King's College London London UK; ^2^ Department of Neurology Bangalore Medical College and Research Institute Bangalore India; ^3^ NIHR‐Wellcome Trust King's Clinical Research Facility King's College Hospital London UK; ^4^ Department of Neurology Taipei Municipal Wanfang Hospital Taipei Taiwan; ^5^ Department of Neurology, School of Medicine, College of Medicine Taipei Medical University Taipei Taiwan; ^6^ Department of Neurology Ghent University Hospital Ghent Belgium; ^7^ Division of Neurology, Department of Medicine National University Hospital, National University Health System Singapore Singapore; ^8^ Yong Loo Lin School of Medicine National University of Singapore Singapore Singapore; ^9^ Department of Neurology University of California Los Angeles California USA

**Keywords:** chronic migraine, medication overuse headache, new daily persistent headache

## Abstract

**Objective:**

To compare the clinical phenotype of patients with chronic migraine (CM) to patients with new daily persistent headache of the chronic migraine subtype (NDPH‐CM).

**Methods:**

A study was conducted of CM (*n* = 257) and NDPH‐CM (*n* = 76) from a tertiary headache center in the UK, and in the US of patients with daily CM (*n* = 60) and NDPH‐CM (*n* = 22).

**Results:**

From the UK cohort, the age of first headache onset was lower in CM (mean ± SD: 16 ± 12 years) than in NDPH‐CM (mean ± SD: 23 ± 14 years; *p* < 0.001). There was a greater number of associated migrainous symptoms in CM compared to NDPH‐CM (median and interquartile range: 6, 5–8 vs. 5, 4–7; *p* < 0.001). A family history of headache was more common in CM compared to NDPH‐CM (82%, 202/248, vs. 53%, 31/59; *p* < 0.001). In the US cohort there were no differences. Osmophobia (*B* = −1.08; *p* = 0.002) and older age at presentation to the clinic (*B* = −0.06; *p* = 0.001) were negative predictors of NDPH‐CM.

**Conclusion:**

NDPH‐CM is relatively less migrainous than CM in the UK cohort. Family history of headache is less common in NDPH‐CM, with negative predictors for NDPH‐CM including osmophobia and older age of presentation to the clinic. More work is required to understand the chronic migraine phenotype of new daily persistent headache.

**Abbreviations**
CGRP: calcitonin gene‐related peptide
CM: chronic migraine
ICHD: International classification of Headache Disorders
IQR: interquartile range
NDPH: new daily persistent headache
NDPH‐CM: NDPH of the chronic migraine subtype

## INTRODUCTION

New daily persistent headache (NDPH) is classified in the International Classification of Headache Disorders, 3rd edition (ICHD‐3) as a primary headache disorder characterized by the clear recollection of the onset of continuous and unremitting headache persisting over the course of a minimum of three months.^1^ The term NDPH may be interpreted literally to go beyond the ICHD‐3 construction as an umbrella diagnosis for a heterogeneous disease category unified by a common temporal profile.^2^, ^3^ It has been stated that its clinical course is refractory.^4^ In previous versions of the ICHD, NDPH was diagnosed only in the absence of migrainous features. Since ICHD‐2, thinking concerning NDPH has evolved.^2^, ^5^ NDPH can have a phenotype similar to migraine or tension‐type headache. It is now widely recognized that the chronic migraine (CM) phenotype of NDPH (NDPH‐CM) comprises the majority of cases of NDPH in headache clinics.^6^ The prevalence of NDPH is challenging to establish; from a population‐based cross‐sectional study of a randomly selected sample of 300,000 persons aged between 30 and 44 years using the stricter ICHD‐2 criteria, it was estimated that the 1‐year prevalence of NDPH was 0.03%.^7^ However, it is likely that the prevalence would be higher using the latest ICHD criteria.

The pathogenesis of NDPH remains unclear. There are theories associating NDPH with viral infections.^8^, ^9^, ^10^ Rozen and Swidan^11^ investigated whether NDPH could result from a persistent state of systemic or central nervous system inflammation. They found an elevation of cerebrospinal fluid tumor necrosis factor alpha levels in 19 out of 20 patients with NDPH; however, this was also found in all of the 16 patients with CM and two patients with post‐traumatic headache who formed the control group. They concluded there is a possible role for cerebrospinal fluid inflammation in both NDPH and treatment‐resistant CM.

The diagnostic feature distinguishing NDPH from CM is the patients' ability to pinpoint a particular day of onset of the continuous headache without any preceding increase in headache frequency and severity. In one study, precipitating events were noted in 47% of the patients, with an infection/flu‐like illness being seen in 22%, a stressful life event in 9%, and a further 9% being triggered by surgical procedures involving intubation. No precipitating event could be recognized in 53% of the patients.^12^ A more recent chart review‐based study identified stressful life events to be more common (20%) compared to infection (10%) as a trigger.^13^

Whether NDPH‐CM exists along a spectrum of acute and persistent migraine or, rather, if this disorder represents a distinct clinical entity, remains to be determined. The objective of this study was to compare the clinical phenotype of patients with NDPH‐CM to that of a large number with CM and daily CM in two tertiary headache centers. Our original hypothesis was that NDPH‐CM is a separate entity from CM. It might have a different clinical phenotype as compared to CM, in addition to differences in the mode of onset and therapeutic response. We wanted to explore if any of the clinical symptoms would serve as a distinguishing factor between the two conditions.

## METHODS

### Study population and design

A study was conducted of the clinical letters of patients presenting to the tertiary headache center at King's College Hospital from 2014 to 2019 (UK cohort). Clinical letters were reviewed on patients seen between 2009 and 2013 at the University of California, San Francisco Headache Center (US cohort). All patients at both sites were seen by at least one doctor and reviewed by the senior author (P.J.G.). The study required no Research Ethics Committee approval as per current UK guidelines (http://www.hra‐decisiontools.org.uk/research/). The study was approved by the UCSF Committee for Human Research in the US (IRB #10‐00020). The requirement for written informed consent was waived.

We selected the records of patients with either a diagnosis of CM as defined by the ICHD‐3,^1^ or with a diagnosis of NDPH also fulfilling a CM diagnosis; this latter group was termed NDPH‐CM. All patients with CM and NDPH‐CM seen in the aforementioned time periods were included in the study. Only those with a co‐existent trigeminal autonomic cephalalgia were excluded from the study. Among the patients diagnosed with CM, a separate subgroup of those with daily headache (daily CM) was also tabulated to compare with NDPH‐CM. All clinical data were collected by headache specialty trained physicians. Based on our use of ICHD appendix diagnostic criteria for tension‐type headache, none were identified.

### Data collection

Data compilation was performed by the authors (K.N., D.W., N.V., J.O., S.W., and H.W.). For each patient, the following information was recorded contemporaneously as the clinical history was taken: the age when presented to the tertiary clinic; sex; headache diagnosis; date of the first visit; headache frequency; headache intensity; age of headache onset; type of pain; location of pain and associated migrainous symptoms consisting of photic hypersensitivity, photic allodynia, phonophobia, osmophobia, cranial allodynia, movement sensitivity, lightheadedness, neck stiffness, internal and external vertigo, and restlessness. In addition, aura symptoms, cranial autonomic symptoms, premonitory and postdromal symptoms (when available), triggers, current and past medication, and presence of medication overuse were also recorded. In the results tables, we separated the symptoms to migrainous, premonitory, and postdrome symptoms. The migrainous symptoms refer to the associated symptoms with the headache regardless of phase.

### Statistical analysis

All data were tabulated in Excel for Windows (2016) and analyzed using SPSS Statistics Version 24.0 for Windows (IBM, Armonk, NY: IBM Corp.). No statistical power calculation was conducted prior to the study. The sample size was based on the available data. It was decided to do a primary analysis of the UK and US data separately as there were differences in the clinical parameters collected in the two cohorts. The data were also collected in different time periods, so we did not pool the data for analysis. For categorical variables, data are summarized as percentages. Descriptive statistics were used and presented as mean ± SD or median with interquartile range (IQR) dependent on the distribution of the data. One‐way analysis of variance and chi‐squared analysis were used for comparisons of continuous and categorical variables, respectively. Bonferroni correction for multiple comparisons was performed, and the adjusted *p* is indicated for both the UK and US data. A post hoc binary logistic regression analysis using a logit link function was performed on the UK data to determine the predictive effect of tabulated variables on the dependent variable: NDPH‐CM versus CM, and NDPH‐CM versus daily CM. Demographic and outcome data were modeled separately from phenotype data as potentially trait and state features. *B*‐value refers to the regression weight. A probability level of *p* < 0.05 was considered significant for the data from the binary logistic regression analysis. In the first logistic regression model, demographic factors (age, age of first headache, sex, family history of headache), and treatment factors (total number of preventives used, medication overuse, and 50% reduction of headache days at follow up) were included. In the second model, clinical features such as associated migrainous symptoms, cranial autonomic symptoms, premonitory and postdromal symptoms, were included in the analysis.

## RESULTS

### Demographic data

Patients diagnosed with CM (*n* = 257) and NDPH‐CM (*n* = 76) were identified in the UK cohort. From the US cohort, we also identified patients with CM (*n* = 92) and NDPH‐CM (*n* = 22; Table 1).

**TABLE 1:** Main demographic and clinical characteristics of NDPH‐CM and CM patient groups in the UK and US cohorts

Parameters	UK cohort			US cohort		
	NDPH‐CM	CM	*p* *	NDPH‐CM	CM	*p* **
Number of patients	*n* = 76	*n* = 257		*n* = 22	*n* = 92	
Age (mean ± SD)	39 ± 14	43 ± 15	0.020	26 ± 15	33 ± 17	0.221
Female:male ratio %	F:M (71%)	F:M (83%)	0.022	F:M (64%)	F:M (76%)	0.282
Age at first headache (mean ± SD)	23 ± 14	16 ± 12	**0.001**	23 ± 13	20 ± 12	0.134
Family history of headache	*n* = 31 (53%; data available for *n* = 59)	*n* = 202 (82%; data available for *n* = 248)	**0.001**	17 (77%)	80 (87%)	0.311
Number of associated migrainous symptoms	5 (IQR: 4–7)	6 (IQR: 5–8)	**0.001**	4 (3–5)	4 (3–5)	0.529
Number of cranial autonomic symptoms	2 (IQR: 0–4)	2 (IQR: 1–4)	0.798	1.5 (0–2)	1 (0–3)	0.935
Number of premonitory symptoms	3 (IQR: 2–4)	3 (IQR: 2–5)	0.182	2 (2–4)	3 (2–5)	0.269
Duration of premonitory symptoms (h)	0 (IQR: 0–2)	8 (IQR: 2–24)	**0.001**	NA	NA	
Number of postdromal symptoms	1 (IQR: 0–2)	2 (IQR: 1–3)	0.008	NA	NA	
Duration of postdromal symptoms (h)	3 (IQR: 0–24)	24 (IQR: 6–48)	**0.001**	NA	NA	
Medication overuse	*n* = 25 (33%)	*n* = 132 (51%)	0.006	3 (13.6%)	38 (41.3%)	0.015
Number of previous preventives tried	7 (IQR: 5–10)	6 (IQR: 4–9)	0.010	NA	NA	
Duration of follow up (months)	21 (IQR: 11–32)	26 (IQR: 13–37)	0.149	NA	NA	
50% reduction in headache days at follow‐up	*n* = 6 (10%)	*n* = 33 (18%)	0.160	NA	NA	
*Associated migrainous symptoms*						
Photic hypersensitivity	52 (74%)	224 (87%)	0.015	18 (81.8%)	82 (89.1%)	0.467
Photic allodynia	15 (21%)	35 (14%)	0.133	NA	NA	
Phonophobia	51(73%)	217 (84%)	0.035	19(86.4%)	78 (84.8%)	˃0.999
Osmophobia	20 (29%)	151 (59%)	**0.001**	12 (54.5%)	49 (53.3%)	˃0.999
Nausea	41 (59%)	210 (82%)	**0.001**	17 (77.3%)	72 (78.3%)	˃0.999
Vomiting	20 (29%)	129 (50%)	**0.002**	NA	NA	
Cranial allodynia	40 (57%)	164 (64%)	0.331	NA	NA	
Movement sensitivity	55 (79%)	219 (85%)	0.201	19 (86.4%)	83 (90.2%)	0.698
Lightheadedness	9 (13%)	60 (23%)	0.069	NA	NA	
Neck stiffness	20 (29%)	102 (40%)	0.096	NA	NA	
Internal vertigo	21 (30%)	62 (24%)	0.353	NA	NA	
External vertigo	14 (20%)	54 (21%)	˃0.999	NA	NA	
Restlessness	7 (10%)	11 (4.3%)	0.076	2 (9.1%)	6 (6.6%)	0.652
*Aura*	28 (40%)	139 (54%)	0.043	5 (22.7%)	26 (28.3%)	0.791
*Cranial autonomic symptoms*						
Lacrimation	23 (33%)	83 (32%)	˃0.999	6 (27.3%)	25 (27.2%)	˃0.999
Conjunctival injection	19 (27%)	56 (22%)	0.342	3 (13.6%)	22 (23.9%)	0.396
Ptosis	12 (17%)	50 (20%)	0.733	3 (13.6%)	16 (17.4%)	˃0.999
Nasal congestion	13 (19%)	89 (35%)	0.009	5 (22.7%)	20 (21.7%)	˃0.999
Ear fullness	23 (33%)	74 (29%)	0.556	6 (27.3%)	17 (18.5%)	0.381
*Premonitory symptoms*						
Mood changes	40 (59%)	147 (57%)	0.890	1 (4.5%)	5 (5.4%)	˃0.999
Concentration difficulty	46 (68%)	167 (65%)	0.775	1 (4.5%)	12 (13%)	0.457
Sleep disturbances	4 (6%)	2 (0.8%)	0.019	NA	NA	
Neck stiffness	21 (31%)	110 (43%)	0.095	2 (9.1%)	16 (17.4%)	0.518
Fatigue	33 (48.5%)	109 (42.4%)	0.410	3 (13.6%)	6 (6.5%)	0.372
Yawning	15 (22.1%)	83 (32.3%)	0.137	2 (9.1%)	10 (10.9%)	˃0.999
*Postdromal symptoms*						
Postdromal irritability	6 (9%)	41 (16%)	0.175	NA	NA	
Lethargy	20 (29.9%)	88 (34.2%)	0.562	NA	NA	
Fatigue	34 (50.7%)	125 (48.6%)	0.785	NA	NA	
Concentration difficulty	14 (20.9%)	65 (25.3%)	0.525	NA	NA	

*Note*: Percentages within the group are shown in brackets. Significant differences are highlighted in bold. Symptoms are shown as the total number per subject. For the complete dataset, see Supporting Information.

Abbreviations: CM, chronic migraine; IQR, interquartile range; NA, not available; NDPH‐CM, new daily persistent headache of the chronic migraine subtype.

* UK cohort: *p* < 0.01 (ANOVA) and *p* < 0.005 (chi‐square tests) are significant

** US cohort: *p* < 0.025 (ANOVA) and *p* < 0.008 (chi‐square tests) are significant.

In the UK cohort, the age of headache onset was significantly less in the CM group versus the NDPH‐CM group (23 ± 14 vs. 16 ± 12; *p* ≤ 0.001). A family history of migraine was more frequent in the CM group compared to the NDPH‐CM group (82% vs. 53%; *p* < 0.001). There was no difference regarding these parameters between the two groups in the US cohort.

### Phenotypic comparison of NDPH‐CM with CM

A previous migraine history was found in 37 (51%) patients of NDPH‐CM in the UK cohort and 17 (77%) in the US cohort. An event immediately preceding the onset of the headache was clearly recalled in 32 (46%) patients in the UK cohort. The time to diagnosis of NDPH‐CM was 5 ± 6 years (mean ± SD) in the UK cohort and 3 ± 4 years in the US cohort.

The mean number of monthly headache days in the CM group was 26 ± 5, with 149 (58%) subjects presenting with continuous pain. By the clinical definition, all subjects in the NDPH‐CM group had continuous pain. When comparing the frequencies of the most common associated migrainous symptoms in the two groups in the UK cohort, significant differences were recorded (Table 1). Notably, the total number of associated migrainous symptoms (median and IQR: 6, 5–8 vs. 5, 4–7; *p* < 0.001) were higher in the CM group. In the CM group, the median duration of premonitory symptoms was 8 h (IQR: 2–24 vs. 0, 0–2; *p* < 0.001), and for postdromal symptoms 24 h (24, 6–48 vs. 3, 0–24; *p* < 0.001); these were longer than in the NDPH‐CM group.

#### Canonical migrainous symptoms

Osmophobia (*n* = 151; 59% vs. *n* = 20; 29%; *p* = 0.001), nausea (*n* = 210; 82% vs. *n* = 41; 59%; *p* ≤ 0.001), and vomiting (*n* = 129; 50% vs. *n* = 20; 29%; *p* = 0.002) were all more frequent in the CM group when compared to the NDPH‐CM group in the UK cohort (Table 1).

#### Cranial autonomic symptoms

There was no significant difference in any of the cranial autonomic symptoms between the CM group and the NDPH‐CM group in either cohort (Table 1).

#### Medications

There was no significant difference between medication overuse in the NDPH‐CM group as compared to the CM group (in the UK cohort: *n* = 25; 33% vs. *n* = 132; 51%: *p* = 0.006). The total number of preventives tried was comparable between the two groups (UK cohort; US cohort data not available). The median follow‐up duration and the percentage of patients with a 50% reduction in headache days were comparable in the two groups (UK cohort; US cohort data not available).

#### Triggers

Alcohol (29% in the NDPH‐CM group vs. 52% in CM group; *p =* 0.001) and the menstrual cycle (10% in the NDPH‐CM group vs. 34% in CM group; *p* = 0.001) were more frequently reported as a trigger for severe headache in the CM group as compared to the NDPH‐CM group in the UK cohort.

### Premonitory and postdromal symptoms

There were no differences between the two groups' frequencies of premonitory and postdromal symptoms in either cohort.

### The US cohort

The exploratory analysis of the US cohort showed that the total number of associated migrainous symptoms was not significantly different between the two groups. Postdromal symptoms were not recorded in the US cohort. There were no significant differences between the NDPH‐CM and CM groups concerning associated migrainous symptoms, aura, cranial autonomic symptoms, or premonitory symptoms (Table 1).

Alcohol and menstrual cycles as trigger factors were no more common in the CM group than the NDPH‐CM group in the US cohort.

Medication overuse was not found to be significantly greater in the CM group in the US cohort following correction for multiple comparisons (*n* = 38; 41.3% vs. *n* = 3;13.6%; *p* = 0.015). There were limited data about the total number of preventives used and therapeutic responses in the US cohort.

### Comparison between NDPH‐CM and daily CM

Of the CM group, 149 patients had daily CM in the UK cohort and 60 patients in the US cohort, respectively (Table 2).

**TABLE 2:** Main demographic and clinical characteristics of daily CM and NDPH‐CM patient groups in the UK and US cohorts

Parameters	UK cohort			US cohort		
	NDPH‐CM	Daily CM	*p* *	NDPH‐CM	Daily CM	*p* **
Number of patients	*n* = 76	*n* = 149		*n* = 22	*n* = 60	
Age (median & IQR)	39 (IQR: 26–48)	42 (IQR: 30–54)	0.125	26 (IQR: 16–45)	29 (IQR: 17–53)	0.506
Female:male ratio %	54:22 (71%)	117:32 (79%)	0.249	F:M (64%)	F:M (78%)	0.253
Age at first headache	19 (IQR: 14–34)	14 (IQR: 9–20)	**0.0001**	23 (IQR: 15–38)	20 (IQR: 13–30)	0.135
Family history of headache	31 (52.5%)	113 (78%)	**0.001**	16 (76%)	51 (87.9%)	0.29
Number of associated migrainous symptoms	5 (IQR: 4–7)	6 (IQR: 5–8)	**0.002**	4 (IQR: 3–5)	4.5 (IQR: 3–5)	0.316
Number of cranial autonomic symptoms	2 (IQR: 0–4)	2 (IQR: 1–4)	0.761	1.5 (IQR: 0–2)	1 (IQR: 0–2)	0.670
Number of premonitory symptoms	3 (IQR: 2–4)	3 (IQR: 1–4)	0.655	2 (IQR: 2–4)	3 (IQR: 2–5)	0.288
Duration of premonitory symptoms (h)	0 (IQR: 0–1.5)	13 (IQR: 0.125–33)	0.082	NA	NA	
Number of postdromal symptoms	1 (IQR: 0–2)	1 (IQR: 1–3)	0.050	NA	NA	
Duration of postdromal symptoms (h)	3 (IQR: 0–24)	24 (IQR: 6–36)	**0.001**	NA	NA	
Medication overuse	*n* = 25 (33%)	*n* = 74 (50%)	0.023	3 (13.6%)	26 (43.3%)	0.018
Number of preventives	7 (IQR: 5–10)	7 (IQR: 4–10)	0.308	NA	NA	
Duration of follow up (months)	21 (IQR: 11–32)	26 (IQR: 17–36)	0.069	NA	NA	
50% reduction of headache days at follow up	6 (10%)	12 (11%)	˃0.999	NA	NA	
*Associated migrainous symptoms*						
Photic hypersensitivity	52 (74%)	132 (89%)	0.010	18 (82%)	54 (90%)	0.446
Photic allodynia	15 (21%)	12 (8%)	0.008	NA	NA	
Phonophobia	51 (73%)	121 (81%)	0.163	19 (87%)	50 (84%)	˃0.999
Osmophobia	20 (29%)	75 (50%)	**0.003**	12 (55%)	34 (57%)	˃0.999
Nausea	41 (59%)	117 (79%)	**0.003**	17 (77%)	49 (82%)	0.755
Vomiting	20 (29%)	71 (48%)	0.008	NA	NA	
Cranial allodynia	40 (57%)	100 (67%)	0.175	NA	NA	
Movement sensitivity	55 (79%)	124 (83%)	0.454	19 (86%)	54 (90%)	0.696
Lightheadedness	9 (13%)	38 (26%)	0.035	NA	NA	
Neck stiffness	20 (29%)	62 (42%)	0.073	NA	NA	
Internal vertigo	21 (30%)	38 (26%)	0.516	NA	NA	
External vertigo	14 (20%)	32 (22%)	0.860	NA	NA	
Restlessness	7 (10%)	7 (5%)	0.147	2 (9%)	5 (8.5%)	˃0.999
*Aura*	28 (40%)	82 (55%)	0.043	5 (23%)	14 (23%)	˃0.999
*Cranial autonomic symptoms*						
Lacrimation	23 (33%)	55 (37%)	0.650	6 (27%)	13 (22%)	0.571
Conjunctival injection	19 (27%)	27 (18%)	0.155	3 (14%)	14 (23%)	0.539
Ptosis	12 (17%)	30 (20%)	0.714	3 (14%)	7 (12%)	˃0.999
Nasal congestion	13 (19%)	47 (32%)	0.052	5 (23%)	7 (12%)	0.289
Ear fullness	23 (33%)	41 (28%)	0.429	6 (27%)	9 (15%)	0.213
*Premonitory symptoms*						
Mood changes	40 (59%)	84 (56%)	0.769	1 (4.5%)	3 (5%)	˃0.999
Concentration difficulty	46 (68%)	94 (63%)	0.544	1 (5%)	9 (15%)	0.275
Food craving	14 (21%)	26 (17%)	0.576	0 (0%)	4 (7%)	0.570
Neck stiffness	21 (31%)	57 (38%)	0.360	2 (9%)	11 (18%)	0.497
Fatigue	33 (49%)	61 (41%)	0.305	3 (13.6%)	5 (8.3%)	0.437
Yawning	15 (22%)	45 (30%)	0.253	2 (9%)	5 (8%)	˃0.999
*Postdromal symptoms*						
Postdromal irritability	6 (9%)	26 (17%)	0.146	NA	NA	
Lethargy	20 (30%)	50 (34%)	0.640	NA	NA	
Fatigue	34 (50.7%)	55 (36.9%)	0.073	NA	NA	
Concentration difficulty	14 (21%)	37 (25%)	0.605	NA	NA	

*Note*: Percentages within the group are shown in brackets. Symptoms are shown as the total number per subject. Only significant differences are shown and highlighted in bold.

Abbreviations: CM, chronic migraine; IQR, interquartile range; NA, not available; NDPH‐CM, new daily persistent headache of the chronic migraine subtype.

* UK cohort: *p* < 0.01 (ANOVA) and *p* < 0.005 (chi‐square tests) are significant

** US cohort: *p* < 0.025 (ANOVA) and *p* < 0.008 (chi‐square tests) are significant.

In the UK cohort, the age of the first headache onset was lower in the daily CM group (mean ± SD: 17 ± 12 vs. 24 ± 14; *p* < 0.0001). A positive family history of headache was more common in the daily CM group (*n* = 113; 78% vs. 31; 52.5%; *p* = 0.001). The total number of associated migrainous symptoms (median and IQR: 6, 5–8 vs. 5, 4–7; *p* = 0.002) was greater in the daily CM group. The duration of postdromal symptoms was greater in the daily CM group (median and IQR: 24, 6–36 vs. 3, 0–24; *p* = 0.001). There were no significant differences regarding these parameters between the two groups in the US cohort. Medication overuse was not significantly different between the two groups, both in the UK and US cohorts.

Among associated migrainous symptoms, osmophobia (*n* = 75; 50% vs. *n* = 20; 29%; *p* = 0.003) and nausea (*n* = 117; 79% vs. *n* = 41; 59%; *p* = 0.003) were significantly more common in the daily CM group as compared to the NDPH‐CM group in the UK cohort (Table 2). None of these parameters significantly differed between the two groups in the US cohort.

### Predictors for NDPH‐CM in the UK cohort

In a binary logistic regression model comparing the demographics and treatment response on the primary outcome of predicting NDPH‐CM or CM (Table 3), and NDPH‐CM or daily CM (Table 4), age of first headache onset was a positive predictor of NDPH‐CM. In contrast, the age of presenting to a tertiary headache clinic was a negative predictor of NDPH‐CM. Collinearity was excluded prior to running the analysis through the use of correlation tables. We found no correlation with *r* > 0.5.

**TABLE 3:** Binary logistic regression analysis of the UK cohort (NDPH CM vs. CM)—Demographics and treatment response

Predictor variable	*B*	*p*	Odds ratio with 95% CI
Age when presented to the tertiary headache clinic	−0.06	**0.001**	0.94 (0.91–0.97)
Age of first headache	0.07	**0.001**	1.07 (1.03–1.11)
Sex	−0.29	0.501	0.75 (0.32–1.74)
Family history of headache	−0.75	0.084	0.47 (0.20–1.11)
Medication overuse	−0.66	0.114	0.52 (0.23–1.17)
Total number of preventives used	0.02	0.728	1.02 (0.92–1.13)
50% reduction in headache days at follow up	−0.62	0.325	0.54 (0.16–1.84)

*Note*: *p* < 0.05 is significant.

Abbreviations: CI, confidence interval; CM, chronic migraine; NDPH‐CM, new daily persistent headache of the chronic migraine subtype.

**TABLE 4:** Binary logistic regression analysis of UK cohort (NDPH CM vs. daily CM)—Demographics and treatment response

Predictor variables	*B*	*p*	Odds ratio with 95% CI
Age when presented to the tertiary headache clinic	−0.06	**0.001**	0.95 (0.92–0.98)
Age of first headache	0.06	**0.003**	1.06 (1.02–1.10)
Sex	−0.16	0.724	0.86 (0.36–2.03)
Family history of headache	−0.52	0.265	0.60 (0.24–1.48)
Medication overuse	−0.60	0.177	0.55 (0.23–1.31)
Total number of preventives	−0.01	0.860	0.99 (0.89–1.11)
50% reduction in headache days at follow up	−0.14	0.842	0.87 (0.23–3.37)

*Note*: *p* < 0.05 is significant.

Abbreviations: CI, confidence interval; CM, chronic migraine; NDPH‐CM, new daily persistent headache of the chronic migraine subtype.

A further logistic regression was performed to ascertain the effects of clinical features on the diagnosis of NDPH‐CM versus CM. Osmophobia and nasal congestion were negative predictors for the diagnosis of NDPH‐CM (Table 5). In a logistic regression model comparing the clinical features between NDPH‐CM with the daily CM cohort, osmophobia and lightheadedness were negative predictors of NDPH‐CM, while photic allodynia but not photic hypersensitivity was a positive predictor of NDPH‐CM (Table 6).

**TABLE 5:** Binary logistic regression analysis of the UK cohort (NDPH CM vs. CM)—Clinical features

Predictor variable	*B*	*p*	Odds ratio with 95% CI
Photic hypersensitivity	−0.20	0.630	0.82 (0.36–1.87)
Photic allodynia	0.51	0.239	1.67 (0.71–3.91)
Phonophobia	−0.34	0.434	0.71 (0.31–1.66)
Osmophobia	−1.08	**0.002**	0.341 (0.171–0.677)
Nausea	−0.48	0.241	0.62 (0.28–1.38)
Vomiting	−0.39	0.330	0.68 (0.31–1.49)
Cranial allodynia	0.04	0.908	1.04 (0.52–2.10)
Movement sensitivity	−0.16	0.712	0.85 (0.37–1.98)
Lightheadedness	−0.81	0.085	0.44 (0.18–1.12)
Neck stiffness	−0.36	0.323	0.70 (0.34–1.42)
Internal vertigo	0.42	0.286	1.52 (0.71–3.27)
External vertigo	0.13	0.762	1.14 (0.49–2.62)
Restlessness	1.10	0.095	3.01 (0.83–10.98)
Aura	−0.21	0.551	0.81 (0.41–1.60)
Lacrimation	0.19	0.608	1.21 (0.59–2.49)
Conjunctival injection	0.60	0.149	1.82 (0.81–4.09)
Ptosis	−0.05	0.917	0.95 (0.37–2.43)
Nasal congestion	−0.91	**0.028**	0.40 (0.18–0.91)
Ear fullness	0.52	0.163	1.68 (0.81–3.49)
Mood changes	0.27	0.474	1.31 (0.63–2.72)
Sleep disturbances	1.40	0.200	4.1 (0.5–34.7)
Premonitory concentration difficulty	−0.01	0.974	0.99 (0.44–2.23)
Premonitory neck stiffness	−0.43	0.271	0.65 (0.30–1.40)
Premonitory fatigue	0.32	0.378	1.37 (0.68–2.77)
Premonitory yawning	−0.21	0.612	0.81 (0.37–1.81)
Postdrome irritability	−0.76	0.213	0.47 (0.14–1.55)
Postdrome lethargy	0.10	0.793	1.11 (0.51–2.43)
Postdrome fatigue	−0.08	0.835	0.93 (0.45–1.92)
Postdrome cognitive impairment	0.08	0.869	1.08 (0.44–2.65)

*Note*: *p* < 0.05 is significant.

Abbreviations: CI, confidence interval; CM, chronic migraine; NDPH‐CM, new daily persistent headache of the chronic migraine subtype.

**TABLE 6:** Binary logistic regression analysis of UK cohort (NDPH CM vs. daily CM)—Clinical features

Predictor variable	*B*	*p*	Odds ratio with 95% CI
Photic hypersensitivity	−0.46	0.364	0.63 (0.24–1.70)
Photic allodynia	1.18	**0.039**	3.24 (1.06–9.92)
Phonophobia	−0.10	0.844	0.91 (0.35–2.36)
Osmophobia	−0.81	**0.041**	0.45 (0.21–0.97)
Nausea	−0.26	0.579	0.78 (0.32–1.91)
Vomiting	−0.39	0.380	0.68 (0.28–1.62)
Cranial allodynia	−0.14	0.731	0.87 (0.39–1.95)
Movement sensitivity	−0.10	0.829	0.90 (0.36–2.26)
Lightheadedness	−1.10	**0.031**	0.333 (0.122–0.907)
Neck stiffness	−0.29	0.474	0.75 (0.34–1.66)
Internal vertigo	0.19	0.660	1.21 (0.52–2.84)
External vertigo	0.36	0.441	1.43 (0.58–3.55)
Restlessness	0.93	0.207	2.53 (0.60–10.75)
Aura	−0.08	0.840	0.93 (0.44–1.96)
Lacrimation	−0.17	0.660	0.84 (0.39–1.81)
Conjunctival injection	0.94	0.052	2.55 (0.99–6.55)
Ptosis	−0.12	0.820	0.89 (0.33–2.40)
Nasal congestion	−0.64	0.165	0.53 (0.21–1.30)
Ear fullness	0.49	0.237	1.63 (0.73–3.68)
Mood changes	0.28	0.532	1.32 (0.56–3.12)
Sleep disturbance	1.16	0.378	3.20 (0.24–42.53)
Premonitory concentration difficulty	−0.22	0.641	0.80 (0.31–2.05)
Premonitory neck stiffness	−0.27	0.530	0.76 (0.32–1.79)
Premonitory fatigue	0.45	0.260	1.57 (0.72–3.42)
Premonitory yawning	−0.23	0.599	0.80 (0.34–1.85)
Postdrome irritability	−0.62	0.326	0.54 (0.16–1.86)
Postdrome lethargy	0.42	0.338	1.53 (0.64–3.61)
Postdrome fatigue	0.47	0.244	1.60 (0.73–3.51)
Postdrome concentration difficulty	−0.15	0.759	0.86 (0.33–2.25)

*Note*: *p* < 0.05 is significant.

Abbreviations: CI, confidence interval; CM, chronic migraine; NDPH‐CM, new daily persistent headache of the chronic migraine subtype.

## DISCUSSION

The presented data suggest that NDPH‐CM has a distinct balance of clinical features compared with CM, even when compared with daily CM. An earlier age of headache onset, a family history of headache, and a higher number of associated migrainous symptoms were more common in patients with CM than NDPH‐CM. Premonitory and postdromal durations were longer in CM than in NDPH‐CM. An older age presenting to the tertiary headache clinic and a younger age of the first headache were predictors for CM and daily CM, with osmophobia being a negative predictor for NDPH‐CM.

In our study, NDPH‐CM is less migrainous. In keeping with this, the durations of premonitory and postdrome symptoms were higher in the CM group than in the NDPH‐CM group. The duration of postdrome symptoms remained lower in NDPH even when compared with the daily CM group. A possible explanation for this could be that recording premonitory and postdromal symptoms and their durations might be skewed by the presence of a constant and unremitting headache from the onset.

ICHD‐3 criteria describe the presence of two subtypes of NDPH, the migraine subtype and the tension‐type headache subtype.^1^ Refining the phenotype is essential to furthering our understanding of the underlying pathology and treatment options. Our study showed a significantly greater number of associated migrainous symptoms in the CM group compared to NDPH‐CM, showing that the latter is less “migrainous.” In contrast, a recent sizeable pediatric study compared 155 patients with NDPH with 986 patients with continuous CM and found few clinical differences between the two groups.^14^ The authors suggested no meaningful differences in disease processes in the youth population they studied but rather differences in the manifestation of continuous CM over time. Lobo and colleagues^15^ studied 162 patients with NDPH, out of whom 89.7% had a chronic migraine phenotype. Thunderclap onset was recorded in 14.8%, and more than one headache phenotype was seen in 15.4%. Prior headache was reported in 53.7%, with the full dataset of the previous history of headache available in 46% of the patients, and only 11.1% had an antecedent trigger. A fifth improved on preventive medication, with a persisting subform being present in 51%. The authors feel that as most of the cases in their cohort were phenotypically similar to CM, with more than half reporting prior history of headache, NDPH may not be a separate entity but rather indicates a mode of onset of a primary headache phenotype. This is also supported by the therapeutic response of NDPH‐CM to migraine preventives. However, the authors did not directly compare NDPH‐CM with CM, and daily CM, as performed here. The authors of a pediatric study^14^ concentrated on migraine‐associated features, such as nausea, vomiting, photophobia, and phonophobia. Similarly, Evans and Turner focused largely on canonical migraine symptoms when comparing a largely (80%) migrainous NDPH cohort.^13^ In contrast, our study explored a wider set of migraine characteristics in an adult population. Therefore, the differences in conclusions could be due to the different age groups and differences in migraine features compared.

Although not statistically significant following Bonferroni correction, it is pertinent to mention there was a trend for medication overuse to be numerically more common in the CM and daily CM groups compared with NDPH‐CM. The pathogenesis of medication overuse is not fully understood; however, it is likely to arise from changes in physiological processes causing upregulation of mediators such as calcitonin gene‐related peptide (CGRP). Alterations in functional connectivity and brain structures on neuroimaging^16^ suggest an underlying biological susceptibility.^17^ Patients with migraine are identified as having a higher risk of developing medication overuse.^18^, ^19^, ^20^ This is compared to other primary headache disorders, such as cluster headache, unless the patient also has a history or family history of migraine.^21^ Prakash and colleagues also reported a low frequency of medication overuse (14%) in their series of patients with NDPH with migrainous features.^22^ A low frequency of medication overuse in patietns with NDPH is interesting as it suggests a lower predisposition for medication overuse in those with NDPH. Whether this may be due to a lack of clinical effect from acute medication in patients with NDPH or it is because they present with continuous pain from the onset, is something which needs clarification in future studies. It is challenging to ascertain whether medication overuse is the main cause of new onset of CM or whether it is a consequence of increased headache frequency.

From our logistic regression analyses, osmophobia was a consistent clinical feature with a negative predictive factor that could help distinguish between NDPH‐CM and the CM and daily CM cohorts. Interestingly photic allodynia, but not photic hypersensitivity, was a positive predictor for NDPH‐CM compared to daily CM. Photic allodynia is defined as migraine pain worsened by the presence of light, and in photic hypersensitivity, light is experienced as bothersome without pain. Worsening of migraine pain is thought to be the substrate of light entering the retina, and via an intact optic nerve, the photic signals travel via the dural nociceptive trigeminothalamic neurons.^23^ Photic hypersensitivity and osmophobia occur because of cortical hyperexcitability; therefore, it is intriguing that only osmophobia was found to be a negative predictor and no other symptoms of cortical hyperexcitability. This highlights the importance of teasing out the different migrainous symptoms as they can allude to different underlying pathophysiologies.

Furthermore, from our logistic regression analyses, an older age at presentation to a tertiary headache clinic and a younger headache presentation were positive predictors for CM and daily CM. This is consistent with our understanding of migraine, where migraine starts in teenage years, and NDPH‐CM patients are seen in tertiary headache clinics earlier than patients with CM and daily CM. A younger presentation to the tertiary headache clinic suggests there is something in the NDPH‐CM presentation that prompts a specialist review compared to CM and daily CM. One rationale could be that NDPH‐CM represents a more severe subgroup with a poorer outcome than the NDPH phenotype with tension‐type characteristics.^6^

Interestingly, despite NDPH‐CM having a poorer outcome, the percentage of patients with a 50% reduction in headache days was comparable in the two groups after a comparable follow‐up duration in our study. This could suggest that some persistence with therapy is needed and perhaps that it takes longer for preventives to have their effect. Our patients were recruited from a tertiary headache center; this would be biased towards a more complex population, refractory to treatment. With the development of targeted migraine treatment, anti‐CGRP pathway monoclonal antibodies, particularly as used in NDPH‐CM, would help get insights into the underlying pathophysiology. The use of anti‐CGRP pathway monoclonal antibodies in chronic refractory headache in the adolescent population has been studied, with the majority of the target cohort having CM, but including twelve NDPH patients, as well as persistent post‐traumatic headache. The authors reported a significant reduction in headache frequency compared to baseline; however, in this study, they did not separately report the effect on each headache type, which would help further our understanding of the pathophysiology of NDPH‐CM.^24^ The New Daily Persistent Headache Biomarkers Study investigates the CGRP levels and nerve growth factor levels in patients with NDPH and patients with CM compared with healthy volunteers.^25^ This study is currently recruiting, and the results will hopefully provide some of the missing data to our understanding. Occipital nerve stimulation is a treatment used for refractory CM. However, a recent study of nine NDPH‐CM patients with occipital nerve stimulation implanted did not have a beneficial effect,^26^ again highlighting the differences between CM and NDPH‐CM.

Hence, our study shows significant differences in multiple clinical features between NDPH‐CM and CM, even when considering a subset of patients with daily CM. This raises the possibility of differences in pathophysiology between the two conditions, perhaps not simply that NDPH‐CM is on the migraine spectrum. It may also be that NDPH‐CM may not be a homogeneous condition and may include diverse pathologies. Clues to the underlying pathologies may lie in clinical details like the mode of onset and the nature of triggering factors.

### Limitations

The key limitation of this study is that it was performed retrospectively, although the clinical data were collected contemporaneously and systematically recorded in the clinical notes by a standard set by one of us. The study was conducted in tertiary headache centers. This might have been biased towards selecting a more refractory group of NDPH patients, not fully representative of the condition itself. It is, in fact, less likely that patients with a self‐limiting subtype would seek this type of care. There were large imbalances between the number of patients in the NDPH‐CM compared with the CM groups, which reflects the prevalence of the conditions; this may have influenced the results. The data were extracted by different people and we did not measure for interrater reliability in this study. Lastly, the division of the analysis in trait and state characteristics for modeling was considered a reasonable attempt to understand the underlying biology. Some aspects ascribed to the trait may well have been state.

We aimed to compare the UK and US cohorts with the hope to discover similar trends between the transatlantic cohorts. However, given the smaller sample size and limited data in terms of clinical symptomatology, the US data were tested as exploratory analyses. Furthermore, the migrainous symptoms reviewed in the US cohort differed significantly from the UK cohort. Data regarding postdromal symptoms were not collected in the US cohort; therefore, they were not entirely comparable.

## CONCLUSIONS

The key differences described in the clinical presentation of NDPH‐CM suggest that this condition, albeit manifesting within the CM phenotype, might be characterized by different pathogenetic elements with respect to *vanilla* CM. What remains unresolved is whether NDPH‐CM is categorically different to CM from a pathophysiological perspective or different by degree only, or indeed if there exists an NDPH‐CM subgroup driving the differences observed. This question can have direct therapeutic implications when drugs specifically designed for migraine, such as monoclonal antibodies acting on the CGRP pathway, are used. According to our hypothesis, there can be a differential response characterized by a better outcome in CM as compared to NDPH‐CM. This hypothesis needs to be tested in future studies.

## AUTHOR CONTRIBUTIONS

*Study concept and design*: Karthik Nagaraj, Diana Y. Wei, Francesca Puledda, Hsing‐Yu Weng, Nicolas Vandenbussche, Jonathan J. Y. Ong, Peter J. Goadsby. *Acquisition of data*: Karthik Nagaraj, Diana Y. Wei, Francesca Puledda, Hsing‐Yu Weng, Sadaf Waheed, Nicolas Vandenbussche, Jonathan J. Y. Ong, Peter J. Goadsby. *Analysis and interpretation of data*: Karthik Nagaraj, Diana Y. Wei, Francesca Puledda, Hsing‐Yu Weng, Sadaf Waheed, Nicolas Vandenbussche, Jonathan J. Y. Ong, Peter J. Goadsby. *Drafting of the manuscript*: Karthik Nagaraj, Diana Y. Wei, Francesca Puledda, Hsing‐Yu Weng, Sadaf Waheed, Nicolas Vandenbussche, Jonathan J. Y. Ong, Peter J. Goadsby. *Revising it for intellectual content*: Karthik Nagaraj, Diana Y. Wei, Francesca Puledda, Hsing‐Yu Weng, Sadaf Waheed, Nicolas Vandenbussche, Jonathan J. Y. Ong, Peter J. Goadsby. *Final approval of the completed manuscript*: Karthik Nagaraj, Diana Y. Wei, Francesca Puledda, Hsing‐Yu Weng, Sadaf Waheed, Nicolas Vandenbussche, Jonathan J. Y. Ong, Peter J. Goadsby.

## CONFLICT OF INTEREST

Dr. Nagaraj reports no conflicts of interest. Dr. Wei reports no conflicts of interest. Dr. Puledda reports no conflicts of interest. Dr. Weng reports no conflicts of interest. Ms. Waheed reports no conflicts of interest. Dr. Vandenbussche has received honoraria from Allergan/Abbvie, Novartis, and Teva Pharmaceuticals. Dr. Jonathan Ong has received honoraria from Novartis, Teva, Lundbeck, and DKSH (Singapore). Dr. Goadsby reports personal fees from Aeon Biopharma, grants and personal fees from Amgen, personal fees from Allergan/Abbvie, personal fees from Biohaven Pharmaceuticals Inc., grants from Celgene, grants and personal fees from CoolTech LLC, personal fees from Dr Reddy's, personal fees from Eli Lilly and Company, personal fees from Epalex, personal fees from GlaxoSmithKline, personal fees from Impel Neuropharma, personal fees from Lundbeck, personal fees from Novartis, personal fees from Pfizer, personal fees from Praxis, personal fees from Sanofi, personal fees from Satsuma, personal fees from Teva Pharmaceuticals, outside the submitted work; in addition, Dr. Goadsby has a patent Magnetic stimulation for headache licensed to eNeura without fee and fees for advice through Gerson Lehrman Group and Guidepoint, and fees for educational materials from UptoDate, Omnia Education, WebMD, and fees for publishing from Oxford University Press, Massachusetts Medical Society, and for medicolegal advice in headache.

## Supporting information


Table S1
