# Supplementary material for: Comparison and predictors of chronic migraine vs. new daily persistent headache presenting with a chronic migraine phenotype
Source: Headache. 2022 Jul 21;62(7):828–38. doi: 10.1111/head.14362 (PMC9545870; doi:10.1111/head.14362)
Supplement: Supplementary file 1 — Table S1 [file HEAD-62-828-s001.docx]

**Supplementary table:** Clinical characteristics of NDPH-CM and CM patient groups in the UK and US cohorts showing recorded and analysed parameters were non-significant. NA= not available.

|  | UK cohort | | | US cohort | | |
| --- | --- | --- | --- | --- | --- | --- |
| Parameters | NDPH-CM | CM | *P* | NDPH-CM | CM | *P* |
| **Associated migrainous symptoms** |  |  |  |  |  |  |
| Blurred vision | 16(22.9%) | 70(27.2%) | 0.541 | NA | NA |  |
| **Cranial autonomic symptoms** |  |  |  |  |  |  |
| Eyelid myokimia | 1(1.4%) | 3(1.2%) | ˃0.999 | NA | NA |  |
| Itchy eyes | 9(12.9%) | 28(10.9%) | 0.672 | 3(13.6%) | 9(9.8%) | 0.698 |
| Periorbital oedema | 7(10%) | 40(15.6%) | 0.336 | 1(4.5%) | 7(7.6%) | ˃0.999 |
| Rhinorrhoea | 11(15.7%) | 48(18.8%) | 0.605 | 2(9.1%) | 9(9.8%) | ˃0.999 |
| Facial flushing | 15(21.4%) | 43(16.8%) | 0.380 | 4(18.2%) | 16(17.4%) | ˃0.999 |
| Facial pallor | 11(15.7%) | 53(20.7%) | 0.400 | 2(9.1%) | 14(15.2%) | 0.733 |
| Facial oedema | 2(2.9%) | 7(2.7%) | ˃0.999 | NA | NA |  |
| Sialorrhoea | 1(1.4%) | 2(0.8%) | 0.517 | NA | NA |  |
| Throat swelling | 4(5.7%) | 6(2.3%) | 0.231 | NA | NA |  |
| **Premonitory symptoms** |  |  |  |  |  |  |
| Head soreness | 0(0%) | 2(0.8%) | ˃0.999 | NA | NA |  |
| Photophobia | 2(2.9%) | 8(3.1%) | ˃0.999 | NA | NA |  |
| Neck stiffness | 21(30.9%) | 110(42.8%) | 0.095 | 2(9.1%) | 16(17.4%) | 0.518 |
| Phonophobia | 2(2.9%) | 3(1.2%) | 0.281 | NA | NA |  |
| Speech disturbance | 1(1.5%) | 8(3.1%) | 0.691 | 0(0%) | 2(2.2%) | ˃0.999 |
| Food craving | 14(20.6%) | 59(23%) | 0.746 | 0(0%) | 6(6.5%) | 0.594 |
| Thirst | 6(8.8%) | 27(10.5%) | 0.823 | 0(0%) | 5(5.4%) | 0.581 |
| Nausea | 0(0%) | 8(3.1%) | 0.212 | 0(0%) | 2(2.2%) | ˃0.999 |
| Flatulence | 0(0%) | 1(0.4%) | ˃0.999 | 0(0%) | 0(0%) |  |
| Diarrhoea | 0(0%) | 7(2.7%) | 0.352 | 0(0%) | 0(0%) |  |
| Constipation | 1(1.5%) | 5(1.9%) | ˃0.999 | NA | NA |  |
| Vomiting | 0(0%) | 0(0%) |  | NA | NA |  |
| Anorexia | 2(2.9%) | 3(1.2%) | 0.281 | NA | NA |  |
| Abdominal pain | 0(0%) | 0(0%) |  | NA | NA |  |
| Changes in micturition | 4(5.9%) | 25(9.7%) | 0.473 | 0(0%) | 1(1.1%) | ˃0.999 |
| Fluid retention | 0(0%) | 0(0%) |  | NA | NA |  |
| Dry mouth | 0(0%) | 2(0.8%) | ˃0.999 | NA | NA |  |
| Hiccups | 1(1.5%) | 0(0%) | 0.209 | NA | NA |  |
| **Postdromal symptoms** |  |  |  |  |  |  |
| Neck stiffness | 4(6%) | 33(12.8%) | 0.134 | NA | NA |  |
| Food cravings | 5(7.5%) | 18(7%) | ˃0.999 | NA | NA |  |
| Food aversion | 0(0%) | 6(2.3%) | 0.352 | NA | NA |  |
| Bowel/bladder changes | 3(4.5%) | 8(3.1%) | 0.703 | NA | NA |  |
